# Salivary gland ultrasonography: a highly specific tool for the early diagnosis of primary Sjögren’s syndrome

**DOI:** 10.1186/s13075-015-0657-7

**Published:** 2015-05-28

**Authors:** Chiara Baldini, Nicoletta Luciano, Gaia Tarantini, Rachele Pascale, Francesca Sernissi, Marta Mosca, Davide Caramella, Stefano Bombardieri

**Affiliations:** Rheumatology Unit, University of Pisa, Via Roma, 67, 56126 Pisa, Italy; Radiology Unit, University of Pisa, Pisa, Italy

## Abstract

**Introduction:**

Recently, a great interest has arisen for salivary gland ultrasonography (SGUS) as a valuable tool for the assessment of major salivary gland involvement in primary Sjögren’s syndrome (pSS. The aims of this study were to test the accuracy of SGUS for the early detection of pSSand to compare the diagnostic performance of SGUS with minor salivary gland biopsy (MSGB) and unstimulated salivary flow (USFR) in this context.

**Method:**

Patients with suspected pSS and symptoms duration of ≤5 years were consecutively enrolled in this study. The diagnosis of pSS was made according to the AECG criteria. SGUS was performed by two radiologists blinded to the diagnosis and a previously reported ultrasound scoring system (De Vita et al. 1992, cut-off ≥ 1) was used to grade the echostructure alterations of the salivary glands. Statistical analysis was performed using SPSS v16.

**Results:**

This study included 50 pSS patients and 57 controls with no-SS sicca symptoms. The mean(SD) age of the pSS group was lower than non-SS group (47(13) vs 53(12)yrs, p = 0.006). No further differences between the two groups were observed. Patients with pSS showed a significantly higher SGUS score in comparison with controls (mean(SD) = 2.1(1.8) vs 0.0(0.4), p = 0.000). The SGUS cut-off ≥ 1 showed a sensitivity (SE) of 66 %, a specificity (SP) of 98 %, a positive predictive value (PPV) of 97 % and a negative predictive value (NPV) of 73 % for pSS diagnosis. The SGUS score correlated also with patients’ MSGB/FS and USFR.

**Conclusions:**

This study confirmed the good performance of SGUS for the early non-invasive diagnosis of pSS. Further research in larger international cohort of patients is mandatory in order to assess the role of SGUS in the diagnostic algorithm of pSS.

## Introduction

Chronic inflammation and progressive dysfunction of salivary and lachrymal glands are among the most distinctive features of primary Sjögren’s syndrome (pSS). Currently, the involvement of salivary glands in pSS is assessed by means of complementary tests such as sialometry, sialoscintigraphy and sialography, in accordance with the American European Consensus Group (AECG) classification criteria. Such tests, added to biopsy of the minor salivary gland (MSGB), may provide valuable information on the anatomical and functional damage in these glands [1]; however, their use in clinical practice is limited by poor specificity for pSS diagnosis. On the other hand, sialography and MSGB are undoubtedly more specific tools but they are quite invasive and may cause distress to the patients [2–4]. Routine histopathological minor salivary gland assessment has important prognostic value [5–7]. However, doubt has recently arisen as to the reliability of MSGB following reports of discrepancies noted across different rheumatology centers [8, 9]. Therefore there is a growing interest in searching for alternative, non-invasive and reliable diagnostic tools potentially able to improve the diagnostic algorithm for pSS [10]. In this context, a number of publications have described convincing findings on the role of salivary gland ultrasonography (SGUS) as an easy, non-invasive, widely available imaging technique for the assessment of salivary gland involvement in pSS [11–14]. In the clinical setting SGUS has good diagnostic properties [15–19]. Yet the value of SGUS in correctly classifying patients with pSS at the very early stages of the disease is still being assessed. More specifically, it is still a matter of debate whether at the onset of the disease, SGUS could eventually replace MSGB in routine clinical practice.

The primary aim of this study therefore, was to test the accuracy of SGUS for the early detection of pSS, particularly in distinguishing pSS from idiopathic sicca syndrome in patients manifesting sicca symptoms for less than five years. The secondary aim of the study was to compare the diagnostic performance of SGUS with MSGB in the early detection of pSS in order to verify whether SGUS might at least limit the indications for MSGB in the clinical setting.

## Methods

### Study design and study population

This single-center, cross-sectional study included patients with suspected pSS, recent onset of dry eyes and dry mouth, and manifestion of sicca symptoms for less than 5 years, and who had presented between January 2011 and March 2013 to the University of Pisa tertiary-care referral centre for pSS. The definitive diagnosis of pSS was made in accordance with the AECG criteria [1]. Patients who did not fulfill the AECG criteria for pSS and received a diagnosis of idiopathic sicca syndrome represented the controls. More specifically, idiopathic sicca syndrome was defined as a condition of persistent, not immune-mediated dry eyes and dry mouth in patients without any systemic disorders potentially responsible for dry eyes and dry mouth (i.e., viral or bacterial infections, sarcoidosis, IgG4 disease, etc.). Patients with secondary SS or subjects presenting with dry mouth and dry eyes in the context of other rheumatic systemic diseases were excluded from the study. All subjects gave informed consent for all procedures, which were carried out in accordance with the principles set in the Declaration of Helsinki. The Institutional Review Board at the University of Pisa, Italy, approved this study.

According to the AECG criteria, a complete work up for pSS was performed in all the patients and controls, including a standardized clinical examination performed by the same rheumatologist, serological and laboratory tests, ocular tests, assessment of the unstimulated salivary flow rate (USFR) and MSGB. The patient information collected included: 1) gender; 2) age at inclusion in the study; 3) history of dry eyes/mouth and/or recurrent parotid enlargement assessed by the AECG questionnaire; 4) duration of subjective sicca symptoms; 5) symptoms/signs suggestive of disease-related extraglandular manifestations, defined according to previous studies, and 6) comorbidities and related treatments. Immunologic tests included: antinuclear antibodies (ANA) determined by indirect immunofluorescence assay on HEp-2 cells (a titer ≥1.160 was considered positive), rheumatoid factor (RF) detected by nephelometry, and antibodies to the extractable nuclear antigens Ro/SS-A and La/SS-B, detected by ELISA. Schirmer’s test and the Lissamine green test were performed and scored as described elsewhere [1]. The USFR was assessed by sialometry. MSGB was graded according to the focusing system [1]. The SGUS examination was performed simultaneously to the other diagnostic procedures being undertaken in all of the 107 subjects.

### Ultrasonography examination

SGUS was performed in all patients by two expert radiologists blinded to the patients’ clinical data. The interobserver reliability was estimated using the kappa index, assessing each of the different US parameters separately. Both the parotid and the submandibular glands were scanned with the patients lying in the supine position with the neck hyper-extended and the head slightly turned to the opposite site. The parotid glands were scanned in both the longitudinal and transverse planes, and the submandibular glands were scanned only in the longitudinal plane. The procedure was performed using the Logiq 9 (General Electric Medical Systems, Milwaukee, WI, USA) equipped with a multifrequency linear probe operated at 10 MHz. The following ultrasound (US) parameters were analyzed and recorded in a predefined form as follows: 1) assessment of gland size (i.e., normal, increased, decreased), parenchymal echogenicity, parenchymal inhomogeneity, and posterior glandular border (visible/non-visible). Parenchymal echogeneity was defined as normal or increased in comparison with the thyroid gland parenchyma and the surrounding soft tissue (muscular structures, subcutaneous fat etc.); 2) changes in the echostructure of the glands were scored according to the scoring system of de Vita et al. [20], which mostly focuses on the inhomogeneity of gland tissue (i.e., the typical abnormal echostructure of the glands), with scores ranging from 0 (i.e., homogenous glands) to 3 (grossly inhomogeneous gland). A mild level of inhomogeneity (score 1) was attributed to isolated hypoechoic areas, while an evident level of inhomogeneity (score 2) was assigned to evident scattered hypoechoic areas with variable size, not uniformly distributed, and/or to multiple punctate or linear non-shadowing densities, and 3) a gross level of inhomogeneity (score 3) was attributed to large round or confluent hypoechoic areas, and/or to linear densities, and/or to multiple cysts or multiple calcifications. The global SGUS score (0–6) was represented by the sum of the scores of each pair of parotid and submandibular glands. If homonymous glands were discordant for the degree of inhomogeneity, the higher degree was considered in assessing the (0–3) single score.

### Statistical analysis

Data were expressed as median (IQR) for continuous variables and as absolute frequencies and percentages for nominal variables. Comparisons were made using the parametric Student’s *t* test and non-parametric Mann-Whitney *U* test, as applicable. Dichotomous variables were compared using contingency table analysis and Fisher’s exact test. Spearman’s rank correlation was used to assess correlation between the SGUS score and different study parameters, including USFR and MSGB focus scores. In all statistical tests, a *p* value <0.05 was considered statistically significant. The interobserver agreement was assessed by using Cohen’s kappa coefficient. Similarly the concordance between US score and different pSS diagnostic tests and procedures was carried out using Cohen’s kappa coefficient. A Cohen’s kappa value >0.70 was considered satisfactory. Statistical analysis was performed by SPSS version 16 (SPSS Inc., Chicago IL, USA).

## Results

### Study cohort

The study cohort consisted of 107 subjects, of whom 50 had definitive pSS and 57 had idiopathic sicca syndrome. Table 1 summarizes the characteristics of the patients. The mean age of the pSS group was lower than the non-pSS group (47 (13) vs 53 (12) yrs, *p* = 0.006). No further differences between the two groups were observed with respect to gender, frequency and duration of dry mouth and dry eye symptoms and objective features of dry eyes.

**Table 1:** Characteristics of the study population

Characteristics	Primary Sjögren’s syndrome (n = 50)	No Sjögren’s syndrome (n = 57)	*P* value
Age, years, mean (SD)	47 (13)	53 (12)	0.006
Symptom duration, mean (SD)	2.3 (1.8)	2.1(1.7)	ns
Female sex, number (%)	48 (96 %)	55 (97 %)	ns
Xerophtalmia, number (%)	44 (88 %)	47 (83 %)	ns
Xerostomia, number (%)	46 (92 %)	49 (86 %)	ns
Abnormal ocular tests, number (%)	37 (74 %)	32 (56 %)	ns
USFR, ml/15 minutes, mean (SD)	2.3 (2.6)	4.8 (3.4)	<0.0001
Focus score, mean (SD)	3.0 (2.3)	0(0)	<0.0001
Hypergammaglobulinemia, number (%)	27 (54 %)	3 (5 %)	<0.0001
Rheumatoid factor, number (%)	26 (52 %)	4 (7 %)	<0.0001
Anti-Ro/SSA, number (%)	30 (60 %)	1 (2 %)	<0.0001
Anti-La/SSB, number (%)	17 (34 %)	none	<0.0001

*USFR* unstimulated salivary flow rate

### Ultrasonography results

#### Salivary gland size and parenchymal echostructure

Salivary gland size was measured for parotid and submandibular glands in pSS patients and subjects with idiopathic sicca syndrome. There was correlation between the gland surface areas on the right and on the left sides and no significant differences were detected in major salivary gland surfaces between the two groups. The interobserver agreement on hypoechogenicity was good, with a Cohen’s kappa value of 0.80. Cohen’s kappa values for homogenicity were: 0.80, 0.70 and 0.90 for grades 1, 2 and 3, respectively. Cohen’s kappa values for glandular size were 0.82 for parotid glands and 0.93 for submandibular glands. Finally, Cohen’s kappa value for the posterior glandular border was 0.73. The echogenicity of the parotid glands was increased in 22/50 pSS patients (44 %) vs 2/57 subjects without pSS (3.5 %), whereas the echogenicity of the submandibular glands was increased in 34/50 of the pSS patients (68 %) vs 4/57 subjects without pSS (7 %). Among the parameters analyzed, inhomogeneity was the most significant in discriminating pSS patients from subjects with idiopathic sicca syndrome. More specifically, abnormal SGUS findings were detected in about 66 % of pSS patients and in fewer than 10 % of controls (*p* <0.0001).

**Fig. 1:**
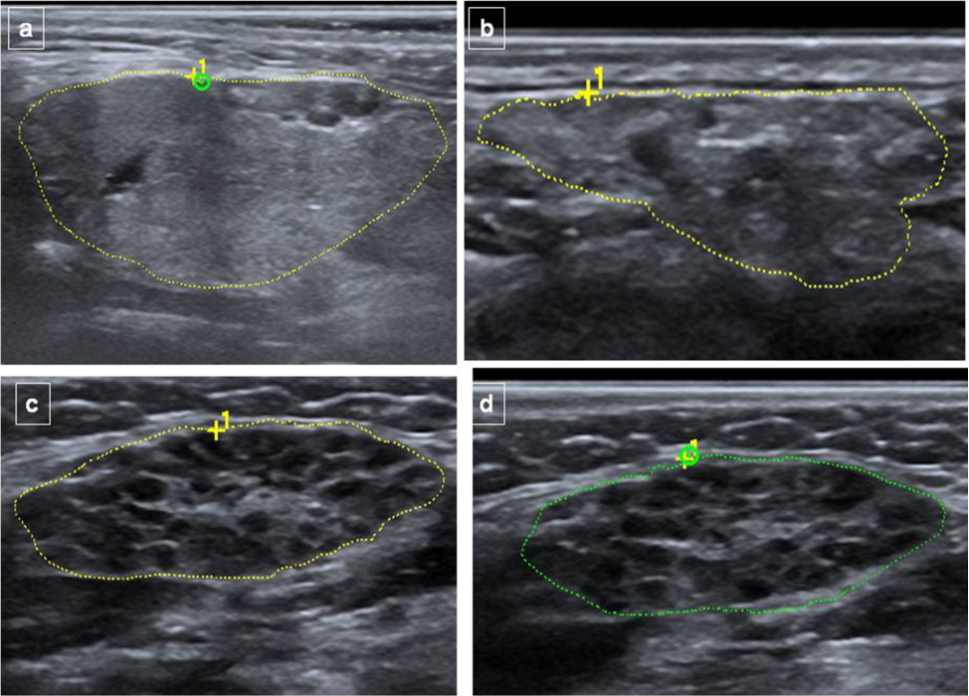
Parenchymal inhomogeneity in the submandibular glands demonstrated by ultrasonography. **a** Normal submandibular gland (grade 0). **b** Submandibular gland with evident inhomogeneity (grade 2). **c**, **d** Submandibular glands with gross inhomogeneity (grade 3)

**Fig. 2:**
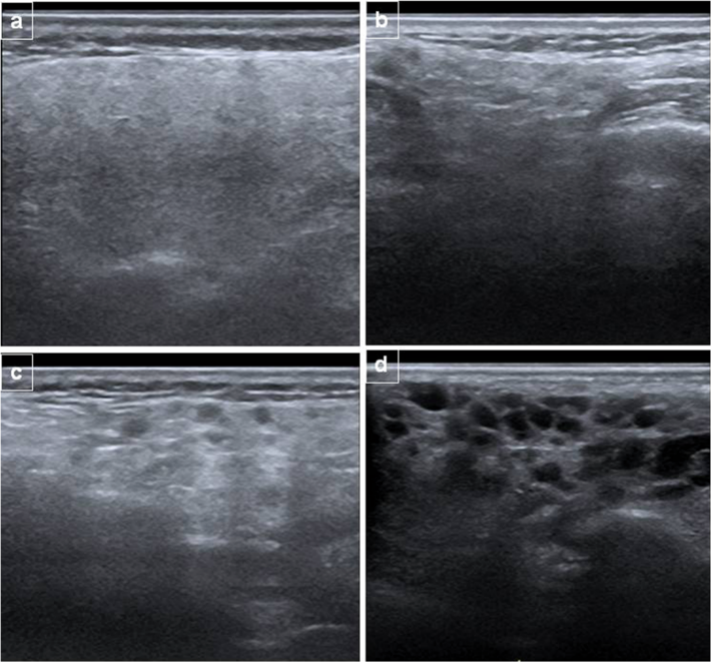
Parenchymal inhomogeneity in the parotid glands demonstrated by ultrasonography. **a** Normal parotid gland (grade 0). **b** Parotid gland with mild unspecific inhomogeneity (grade 1). **c** Parotid gland with evident inhomogeneity (grade 2). **d** Parotid gland with gross inhomogeneity (grade 3)

Table 2 summarizes the inhomogeneity scores for the parotid and submandibular glands in patients with and without pSS. Examples of the parenchymal inhomogeneity grades of both parotid and submandibular glands in patients with pSS and in controls are shown in Figs. 1 and 2. Concordance between the parotid and the submandibular ultrasonographic grades was high with a Cohen’s kappa value of 0.764. The overall SGUS score was significantly higher in pSS patients compared to subjects with idiopathic sicca syndrome (2.1 (1.8) vs 0.1 (0.4), *p* <0.0001). The SGUS score correlated inversely with the USFR (*r* = −0.37, *p* <0.0001) and directly with the MSGB focus score (*r* = 0.39, *p* <0.0001). More specifically, the SGUS score was significantly higher in patients with a USFR <1.5 ml/15 minutes (2.6 ± 1.7 vs 0.9 ± 1.5, *p* <0.0001) and in patients with an MSGB FS ≥1 (2.0 ± 1.8 vs 0.1 ± 0.7, *p* <0.0001).

**Table 2:** Prevalence of SGUS scores in patients with and without primary Sjögren’s syndrome

	Primary Sjögren’s syndrome (n = 50)	No Sjögren’s syndrome (n = 57)	*P* value
SGUS (parotid glands)			
Score 0	17/50 (34 %)	52/57 (91.2 %)	<0.0001
Score 1	15/50 (30 %)	5/57 (8.8 %)	<0.0001
Score 2	15/50 (30 %)	none	<0.0001
Score 3	3/50 (6 %)	none	<0.0001
SGUS (submandibular glands)			
Score 0	23/50 (46 %)	56/57 (98.2 %)	<0.0001
Score 2	24/50 (48 %)	1/57 (1.8 %)	<0.0001
Score 3	3/50 (6 %)	0/57	<0.0001

*SGUS* salivary gland ultrasonography score

#### Diagnostic value of salivary gland echostructure

**Fig. 3:**
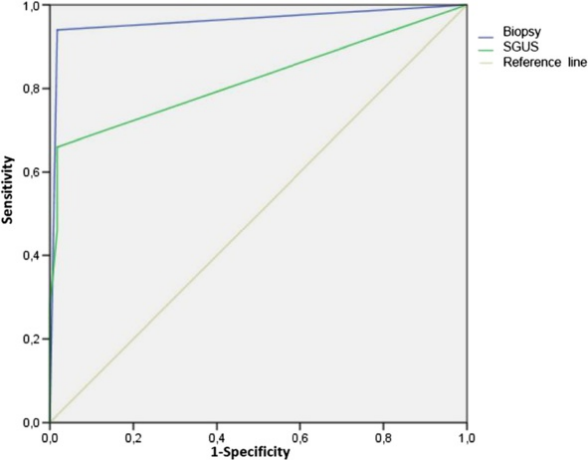
Receiver operator characteristic curves show the diagnostic accuracy of the salivary gland ultrasonography (*SGUS*) score assigned to the parenchymal inhomogeneity (area under the curve (AUC) = 0.82, 95 % CI 0.74, 0.91) and of the minor salivary gland biopsy (AUC = 0.96, 95 % CI 0.92, 1) for primary Sjögren’s syndrom

Consistent with previous studies [13, 20] a cutoff of 2 for the SGUS score allowed us to obtain the best ratio between sensitivity and specificity. The cutoff of 2 was associated with 66 % sensitivity, 98 % specificity, 97 % positive predictive value (PPV) and 77 % negative predictive value (NPV). Table 3 summarizes the diagnostic accuracy of the SGUS in comparison to anti-Ro/SSA, Schirmer’s test and the MSGB focus score. With a cutoff of 2 the diagnostic performance of SGUS was very similar to that observed for the presence of anti-Ro/SSA antibodies in terms of fairly good sensitivity and high specificity: The SGUS score was less sensitive, but more specific than Schirmer’s test. In comparison to MSGB, the SGUS had similar specificity but lower sensitivity, with moderate agreement between the two procedures (kappa Cohen’s value = 0.573). The PPV of the SGUS was higher than for the MSGB alone, which means that when the SGUS results were positive, 33/34 pSS patients (97 %) had been correctly classified. The probability of correctly classifying patients as having pSS was even higher than with the MSGB alone, as only 30/34 pSS patients (88 %) were correctly classified on the basis of the MSGB alone. On the other hand, the NPV of the SGUS score was lower; therefore the probability of correctly classifying the subjects as not having pSS when the SGUS results were negative was only 77 % (i.e., 56/73 subjects with idiopathic sicca syndrome were correctly classified). As a consequence 17/73 pSS patients would have been misclassified on the basis of the SGUS alone without the MSGB. Figure 3 represents the receiver operator characteristic (ROC) curves showing the diagnostic accuracy of the SGUS score (range 0–6) assigned to the parenchymal inhomogeneity (area under the curve (AUC) = 0.82, 95 % CI 0.74, 0.91) and of the MSGB (AUC = 0.96, 95 % CI 0.92, 1) for pSS.

**Table 3:** Diagnostic accuracy of salivary gland ultrasonography (cutoff score of 2), in comparison with anti-Ro/SSA antibodies, Schirmer’s test and minor salivary gland biopsy focus score

	Sensitivity	Specificity	Positive predictive value	Negative predictive value
SGUS ≥2	33/50 (66 %)	56/57 (98 %)	33/34 (97 %)	56 /73 (77 %)
Anti-Ro/SSA antibodies	30/50 (60 %)	56/57 (98 %)	30/31 (97 %)	56/67 (74 %)
Schirmer’s test	37/50 (74 %)	32/57 (56 %)	37/62 (60 %)	32/45 (71 %)
MSGB	46/50 (92 %)	56/57 (98 %)	46/47 (98 %)	56/60 (93 %)

*SGUS* salivary gland ultrasonography score, *MSGB* minor salivary gland biopsy

## Discussion

This study aimed to assess the diagnostic accuracy of SGUS for the early detection of pSS. Indeed, our findings confirmed the potential of SGUS to distinguish pSS from idiopathic sicca syndrome with good sensitivity and high specificity, even at the early stages of the disease. To date, only a limited number of studies have explored the diagnostic value of SGUS for the early detection of pSS. In fact, Cornec et al. [11] investigated the diagnostic accuracy of SGUS in 84 patients with pSS with disease duration ≤5 years, and reported sensitivity of 65.8 % and a specificity of 95.3 %, which is similar to our findings. Other authors have also investigated the performance of SGUS in patients with suspected pSS upon diagnosis of the disease, but did they did not assess the duration of the sicca symptoms prior to the diagnosis [15, 18, 19, 21–28].

Conversely, our study specifically limited enrollment to patients at the onset of the disease. Hence, rather than addressing the duration of the disease, we focused on the duration of the symptoms reported by the patients and developed an ad hoc patient questionnaire. This allowed us to specifically study only patients complaining of sicca symptoms for <5 years in order to demonstrate the accuracy of SGUS for diagnosis of pSS at the early stages of disease in these patients. Intriguingly, by using a scoring system mainly focused on salivary gland inhomogeneity, we were able to demonstrate that changes in the salivary gland parenchymal echostructure appeared relatively early in the course of the disease, with good concordance between parotid and submandibular findings. In line with these data and despite the different scoring systems adopted, all the available studies have highlighted parenchymal gland inhomogeneity as the most important distinctive feature for differentiating pSS from other salivary gland disorders [17]. The SGUS score for inhomogeneity was also the parameter in which interobserver agreement between the radiologists was the highest, reinforcing the reliability and the feasibility of SGUS in daily clinical practice. In addition, the SGUS score was significantly correlated with both the USFR and the MSGB focus score. Therefore, SGUS appeared to mirror dysfunction of the salivary glands and inflammation even in the early stage of the disease. The agreement between SGUS and MSGB was not surprising, because Vitali et al. [29] demonstrated a good level of concordance between minor and major salivary gland involvement in pSS. However, from a practical point of view, our findings demonstrated that SGUS and minor salivary gland histopathology were not interchangeable. More specifically, we speculated that when the SGUS results were negative, MSGB should remain mandatory in order to avoid patient misclassification. By contrast, in patients who have declined biopsy but have a positive SGUS score this may be helpful in routine care. In our study, 33 out of 107 patients (31 %) with pathological findings at the SGUS could have been correctly diagnosed as having pSS without assessment of the MSGB, thus, additionally supporting the value of SGUS for non-invasive diagnosis of the disease.

Despite these encouraging results, our study did have some limitations. First, we used a SGUS score that simply distinguished glands with normal parenchyma from those with moderate or gross changes in the echostructure homogeneity. Yet, other scoring systems available are also able to distinguish between mild and moderate alterations in the salivary gland architecture, and even define the number and size of the hypoechogenic areas detected and the density of the echogenic bands within the glands [19, 27, 30]. These scoring systems might further ameliorate the diagnostic accuracy of SGUS for the early detection of pSS in clinical practice. Moreover, in our study we did not perform Doppler analysis. Actually, contrasting data have been reported on the usefulness of Doppler analysis for the diagnosis of pSS [11, 21]. Recently, Cornec D. et al. [11] showed that Doppler waveform analysis and gland size measurement had poor diagnostic performance when compared to salivary gland inhomogeneity. In this scenario, the international attempt to elaborate a novel common SGUS scoring system will undoubtedly improve the possibility of using SGUS for routine assessment of pSS.

Another limitation of our study in assessing the relationship between SGUS findings and salivary gland dysfunction is that we assessed the entire salivary flow rather than assessing parotid and submandibular salivary fluids separately. Therefore, we could not directly compare parotid and submandibular ultrasonography findings with the respective alterations in major salivary gland secretory function. However, the overall concordance between parotid and submandibular involvement allowed us to demonstrate significant correlation between SGUS findings and salivary flow rate abnormalities.

## Conclusion

Our study represents an additional contribution to the amount of the data supporting the use of SGUS for non-invasive diagnosis of pSS, even in the early stages of the disease. In the present scenario, where the international community is striving to define more specific and sensitive classification criteria for pSS, our results (once validated externally) may further evidence the usefulness and the opportunity of including SGUS among the tests for the diagnosis and the classification of the disease.

## Abbreviations

AECG: American European Consensus Group
ANA: antinuclear antibodies
ELISA: enzyme-linked immunosorbent assay
MSGB/FS: minor salivary gland biopsy focus score
NPV: negative predictive value
PPV: positive predictive value
pSS: primary Sjögren’s syndrome
RF: rheumatoid factor
ROC: receiver operator characteristic
SE: sensitivity
SGUS: salivary gland ultrasonography
SP: specificity
USFR: unstimulated salivary flow rate
US: ultrasound

## References

[1] Vitali C, Bombardieri S, Jonsson R, Moutsopoulos HM, Alexander EL, Carsons SE (2002). Classification criteria for Sjogren’s syndrome: a revised version of the European criteria proposed by the American-European Consensus Group. Ann Rheum Dis.

[2] Jensen SB, Vissink A (2014). Salivary Gland Dysfunction and Xerostomia in Sjogren’s Syndrome. Oral Maxillofac Surg Clin North Am.

[3] Kalk WW, Vissink A, Spijkervet FK, Bootsma H, Kallenberg CG, Nieuw Amerongen AV (2001). Sialometry and sialochemistry: diagnostic tools for Sjogren’s syndrome. Ann Rheum Dis.

[4] Baldini C, Gallo A, Perez P, Mosca M, Alevizos I, Bombardieri S (2012). Saliva as an ideal milieu for emerging diagnostic approaches in primary Sjogren’s syndrome. Clin Exp Rheumatol.

[5] Theander E, Vasaitis L, Baecklund E, Nordmark G, Warfvinge G, Liedholm R (2011). Lymphoid organisation in labial salivary gland biopsies is a possible predictor for the development of malignant lymphoma in primary Sjogren’s syndrome. Ann Rheum Dis.

[6] Carubbi F, Alunno A, Cipriani P, Di Benedetto P, Ruscitti P, Berardicurti O (2014). Is minor salivary gland biopsy more than a diagnostic tool in primary Sjogrens syndrome? Association between clinical, histopathological, and molecular features: a retrospective study. Semin Arthritis Rheum.

[7] Jonsson MV, Theander E, Jonsson R (2012). Predictors for the development of non-Hodgkin lymphoma in primary Sjogren’s syndrome. Presse Med.

[8] Tavoni AG, Baldini C, Bencivelli W, Cavazzini L, Covelli M, De Vita S (2012). Minor salivary gland biopsy and Sjogren’s syndrome: comparative analysis of biopsies among different Italian rheumatologic centers. Clin Exp Rheumatol.

[9] Guellec D, Cornec D, Jousse-Joulin S, Marhadour T, Marcorelles P, Pers JO (2013). Diagnostic value of labial minor salivary gland biopsy for Sjogren’s syndrome: a systematic review. Autoimmun Rev.

[10] Gallo A, Baldini C, Teos L, Mosca M, Bombardieri S, Alevizos I (2012). Emerging trends in Sjogren’s syndrome: basic and translational research. Clin Exp Rheumatol.

[11] Cornec D, Jousse-Joulin S, Pers JO, Marhadour T, Cochener B, Boisrame-Gastrin S (2013). Contribution of salivary gland ultrasonography to the diagnosis of Sjogren’s syndrome: toward new diagnostic criteria?. Arthritis Rheum.

[12] Vitali C, Carotti M, Salaffi F (2013). Is it the time to adopt salivary gland ultrasonography as an alternative diagnostic tool for the classification of patients with Sjogren’s syndrome? Comment on the article by Cornec et al.. Arthritis Rheum.

[13] Theander E, Mandl T (2014). Primary Sjogren’s syndrome: diagnostic and prognostic value of salivary gland ultrasonography using a simplified scoring system. Arthritis Care Res.

[14] Carotti M, Ciapetti A, Jousse-Joulin S, Salaffi F (2014). Ultrasonography of the salivary glands: the role of grey-scale and colour/power Doppler. Clin Exp Rheumatol.

[15] Salaffi F, Carotti M, Iagnocco A, Luccioli F, Ramonda R, Sabatini E (2008). Ultrasonography of salivary glands in primary Sjogren’s syndrome: a comparison with contrast sialography and scintigraphy. Rheumatology.

[16] Wernicke D, Hess H, Gromnica-Ihle E, Krause A, Schmidt WA (2008). Ultrasonography of salivary glands – a highly specific imaging procedure for diagnosis of Sjogren’s syndrome. J Rheumatol.

[17] Tzioufas AG, Moutsopoulos HM (2008). Ultrasonography of salivary glands: an evolving approach for the diagnosis of Sjogren’s syndrome. Nat Clin Pract Rheumatol.

[18] Niemela RK, Takalo R, Paakko E, Suramo I, Paivansalo M, Salo T (2004). Ultrasonography of salivary glands in primary Sjogren’s syndrome. A comparison with magnetic resonance imaging and magnetic resonance sialography of parotid glands. Rheumatology.

[19] Milic VD, Petrovic RR, Boricic IV, Radunovic GL, Pejnovic NN, Soldatovic I (2010). Major salivary gland sonography in Sjogren’s syndrome: diagnostic value of a novel ultrasonography score (0–12) for parenchymal inhomogeneity. Scand J Rheumatol.

[20] De Vita S, Lorenzon G, Rossi G, Sabella M, Fossaluzza V (1992). Salivary gland echography in primary and secondary Sjogren’s syndrome. Clin Exp Rheumatol.

[21] Carotti M, Salaffi F, Manganelli P, Argalia G (2001). Ultrasonography and colour doppler sonography of salivary glands in primary Sjogren’s syndrome. Clin Rheumatol.

[22] Corthouts B, De Clerck LS, Francx L, De Schepper A, Vercruysse HA, Stevens WJ (1991). Ultrasonography of the salivary glands in the evaluation of Sjogren’s syndrome. Comparison with sialography. J Belge Radiol.

[23] Decuzzi M, Tatulli F, Giampaolo M, Tesse R, Gasparre M, Pepe G (2006). Sialocintigraphy versus ultrasonography of the salivary glands in patients first diagnosed with Sjogren’s syndrome. Hell J Nucl Med.

[24] Makula E, Pokorny G, Rajtar M, Kiss I, Kovacs A, Kovacs L (1996). Parotid gland ultrasonography as a diagnostic tool in primary Sjogren’s syndrome. Br J Rheumatol.

[25] Mandel L, Orchowski YS (1998). Using ultrasonography to diagnose Sjogren’s syndrome. J Am Dent Assoc.

[26] Milic V, Petrovic R, Boricic I, Radunovic G, Marinkovic-Eric J, Jeremic P (2012). Ultrasonography of major salivary glands could be an alternative tool to sialoscintigraphy in the American-European classification criteria for primary Sjogren’s syndrome. Rheumatology.

[27] Salaffi F, Argalia G, Carotti M, Giannini FB, Palombi C (2000). Salivary gland ultrasonography in the evaluation of primary Sjogren’s syndrome. Comparison with minor salivary gland biopsy. J Rheumatol.

[28] Takagi Y, Kimura Y, Nakamura H, Sasaki M, Eguchi K, Nakamura T (2010). Salivary gland ultrasonography: can it be an alternative to sialography as an imaging modality for Sjogren’s syndrome?. Ann Rheum Dis.

[29] Vitali C, Tavoni A, Simi U, Marchetti G, Vigorito P, d’Ascanio A (1988). Parotid sialography and minor salivary gland biopsy in the diagnosis of Sjogren’s syndrome. A comparative study of 84 patients. J Rheumatol.

[30] Jousse-Joulin S, Devauchelle-Pensec V, Morvan J, Guias B, Pennec Y, Pers JO (2007). Ultrasound assessment of salivary glands in patients with primary Sjogren’s syndrome treated with rituximab: quantitative and Doppler waveform analysis. Biologics.

